# Consultant psychiatrists’ experience of the impact of the COVID-19 pandemic on mental health services

**DOI:** 10.1017/ipm.2021.41

**Published:** 2021-04-29

**Authors:** E. Kelleher, E. H. Geary, M. Tawfik, E. Ní Mhuircheartaigh, B. Gavin, M. Wall, J. P. Lyne, A. M. Doherty, F. McNicholas

**Affiliations:** 1 Liaison Psychiatry Service, Cork University Hospital, Cork, Ireland; 2 Liaison Psychiatry Service, Mercy University Hospital, Cork, Ireland; 3 Department of Psychiatry, School of Medicine, University College Cork, Cork, Ireland; 4 St Michael’s Unit, Mercy University Hospital, Cork, Ireland; 5 Department of Child & Adolescent Psychiatry, School Medicine and Medical Science, University College Dublin, Belfield, Dublin, Ireland; 6 Acute Mental Health Unit, Cork University Hospital, Cork, Ireland; 7 Wicklow Mental Health Services, Newcastle Hospital, Greystones, Co. Wicklow, Ireland; 8 Royal College of Surgeons in Ireland, Dublin, Ireland; 9 Liaison Psychiatry Service, Mater Misericordiae University Hospital, Dublin, Ireland; 10 Department of Psychiatry, University College Dublin, Dublin, Ireland; 11 Lucena Child and Adolescent Mental Health Services, St John of Gods, Rathgar, Dublin, Ireland; 12 Children’s Health Ireland, Crumlin, Dublin, Ireland

**Keywords:** Consultant experience, COVID-19, mental health

## Abstract

**Objectives:**

The novel coronavirus 2019 (COVID-19) has spread worldwide threatening human health. To reduce transmission, a ‘lockdown’ was introduced in Ireland between March and May 2020. The aim of this study is to capture the experiences of consultant psychiatrists during lockdown and their perception of it’s impact on mental health services.

**Methods:**

A questionnaire designed by the Royal College of Psychiatrists was adapted and circulated to consultant members of the College of Psychiatrists of Ireland following the easing of restrictions. The questionnaire assessed the perceived impact on referral rates, mental health act provision, availability of information technology (IT), consultant well-being and availability of personal protective equipment (PPE). Thematic analysis was employed to analyse free-text sections.

**Results:**

Response rate was 32% (*n* = 197/623). Consultants reported an initial decrease/significant decrease in referrals in the first month of lockdown (68%, *n* = 95/140) followed by an increase/significant increase in the second month for both new (83%, *n* = 100/137) and previously attending patients (65%, *n* = 88/136). Social isolation and reduced face-to-face mental health supports were among the main reasons identified. The needs of children and older adults were highlighted. Most consultants (76%, *n* = 98/129) felt their working day was affected and their well-being reduced (52%, *n* = 61/119). The majority felt IT equipment availability was inadequate (67%, *n* = 88/132). Main themes identified from free-text sections were service management, relationship between patients and healthcare service and effects on consultants’ lives.

**Conclusions:**

The COVID-19 pandemic has placed increased pressure on service provision and consultant wellness. This further supports the longstanding need to increase mental health service investment.

## Background

Since its identification in December 2019, severe acute respiratory syndrome coronavirus 2 (SARS-CoV-2) commonly referred to as ‘COVID-19’ has moved swiftly through the world causing a global pandemic. The first community acquired case in Ireland was identified in February 2020 (Faller *et al.* 2020) with the World Health Organisation declaring a pandemic on 11th March 2020. Overall 6.4% of probable and possible cases had died in Ireland due to COVID-19 by 18th May 2020 (https://covid19ireland-geohive.hub.arcgis.com).

Due to the acceleration of the death rate from coronavirus, the government declared a ‘stay-at-home’ order or ‘lockdown’ on 27th March 2020, with individuals advised to stay at home insofar as possible and to only exercise/move within a 2-km radius of their home. The effect of these measures impacted every aspect of daily life. Day centres, day hospitals, public health nurses and community nurses either stopped providing services or only provided services in a very limited format. Face-to-face outpatient clinics and general practice provision was curtailed and shifted rapidly to providing telemedicine assessments. Hospitals and nursing homes were closed to visitors unless exceptional/compassionate grounds. The lockdown officially lasted from 27th March to 18th May 2020 when the government published a ‘roadmap’ to the easing of restrictions (Department of the Taoiseach, 2020).

On 15th April 2020, the Royal College of Psychiatrists (RCPsych) began conducting a series of surveys of consultant psychiatrist members working in the United Kingdom (UK) on the impact of COVID-19 on local psychiatric services, including on rates of referral, well-being of psychiatrists, and availability of personal protective equipment (PPE). 11% (*n* = 1,369/12,900) of consultants responded to the second survey which focused on the change in demand for services. 43% of those respondents who answered questions about referral rates (*n* = 501/1,177) reported increased workload for urgent and emergency presentations in the fortnight prior to completing the survey (Royal College of Psychiatrists, 2021). Reports in the media at the time quoted the then president of the college Professor Wendy Burn expressing concern that ‘the lockdown is storing up problems which could then lead to a tsunami of referrals’ (Roxby, 2020).

The aim of the current study is to capture the experience of consultant psychiatrists working in Ireland during this COVID-19 lockdown and its impact on psychiatry services during this time.

## Methods

The survey questionnaire was initially developed by the Royal College of Psychiatrists. The study authors adapted this questionnaire with permission. It examined the impact of COVID-19 in several important areas namely: (1) delivery of clinical services; (2) mental health act (MHA) provision; (3) working day of consultant psychiatrists; (4) availability of information technology (IT) equipment; (5) well-being of consultants; and (6) personal protective equipment (PPE). The questionnaire included both quantitative responses and free-text sections in these areas. An arbitrary timepoint of 1 month (4 weeks) was chosen to delineate between the early and later part of the lockdown, for example, between the first month, 27th March 2020 to 24th April 2020, and the second month, 24th April 2020 to 22nd May 2020.

The authors liaised closely with the College of Psychiatrists of Ireland for dissemination of the questionnaire. Following ethical approval, via the ethics board at University College Cork, the questionnaire was uploaded to a surveymonkey platform and circulated to the consultant members of the College of Psychiatrists of Ireland (CPI) membership list via email on 29th May 2020. Consultants were working across a range of settings, both in community healthcare organisation (CHO), voluntary hospitals and private practise. Reminder emails were circulated over the next 10 days. Results were analysed with descriptive statistics using SPSS software.

A thematic analysis was performed on free-text sections of 479 individual statements following the Braun and Clark model (2006) by E.K., M.W., M.T. and E.G. This process follows six steps namely: (1) becoming familiar with the data; (2) generating initial codes; and (3) searching for themes. At this stage, codes had been organised into broader themes that said something specific. The division of the questionnaire into sections guided this process. The authors reviewed (step 4) and defined themes and subthemes (step 5), identifying quotes that were congruent with the key themes before writing up (Step 6). The authors (E.K., M.W., M.T., E.G.) moved between these steps given the complex nature and volume of data.

## Results

The response rate was 32% (*n* = 197/623), although not all respondents answered every question. The most common study participant demographic was female, aged 50–59 years old, working in public service as a general adult psychiatrist. Most respondents were working in CHO areas in Dublin [(CHO) 7 (20%), CHO9 (16%), CHO6 (14%)] and Cork/Kerry CHO4 (15%). See Table 1.

**Table 1.** Demographics of respondents

	% (*n* = 197)
Gender	
Female	62% (*n* = 122)
Male	37% (*n* = 73)
Prefer not to say	1% (*n* = 2)
Age	
Under 30 years old	0
30–39 years old	8% (*n* = 16)
40–49 years old	31% (*n* = 62)
50–59 years old	41% (*n* = 80)
>60 years old	18% (*n* = 36)
Prefer not to say	2% (*n* = 3)
Designated specialty	
General adult psychiatrist	49% (*n* = 97)
Child and adolescent psychiatrist	18% (*n* = 35)
Psychiatrist of later life	14% (*n* = 27)
Liaison psychiatrist	7% (*n* = 14)
Psychiatrist of intellectual disability	7% (*n* = 14)
Academic-clinical psychiatrist	6% (*n* = 12)
Social and rehabilitation psychiatrist	5% (*n* = 10)
Psychiatrist in home based care team	3% (*n* = 5)
Psychiatrist of eating disorders	2% (*n* = 4)
Addiction psychiatrist	2% (*n* = 4)
Perinatal psychiatrist	2% (*n* = 3)
Psychiatrist in student mental health	2% (*n* = 3)
Medical psychotherapist	1% (*n* = 1)
Psychiatrist in early intervention in psychosis	1% (*n* = 2
Psychiatrist in HBCT/EIP	1% (*n* = 2)
Other	6% (*n* = 11)
Area of work	
CHO7	20% (*n* = 39)
CHO9	16% (*n* = 32)
CHO4	15% (*n* = 29)
CHO6	14% (*n* = 27)
Private practice (outside of a private hospital)	10% (*n* = 20)
CHO5	9% (*n* = 17)
CHO1	9% (*n* = 18)
CHO2	8% (*n* = 16)
CHO8	8% (*n* = 16)
CHO3	6% (*n* = 12)
Private hospital	6% (*n* = 12)
Central mental hospital	2% (*n* = 3)
Other	1% (*n* = 1)

### Impact on referral rate

The majority of consultants (68%, *n* = 95/140) experienced a decrease/significant decrease in the number of new referrals in the month following the lockdown. During the second month, the majority of respondents (83%, *n* = 100/137) identified the number of new referrals had increased/significantly increased compared to the first month (Table 2). Over a third of respondents (35%, *n* = 48/136) felt that the number of new referrals had increased/significantly increased compared to before the lockdown.

**Table 2.** Impact of COVID-19 lockdown on relapses and new referrals to secondary mental health services

In the month after full restrictions on movement came into effect (27th March–24th April full lockdown) did this affect the number of referrals for secondary mental health services that you provide?					
Significantly increased	Increased	No difference	Decreased	Significantly decreased	N/A
1% (*n* = 2)	7% (*n* = 10)	24% (*n* = 33)	48% (*n* = 67)	20% (*n* = 28)	
In the second month (24th April–22nd May) compared to the first month of lockdown (27th March–24th April) has the number of new referrals for secondary mental health services that you provide?					
Significantly increased	Increased	No difference	Decreased	Significantly decreased	N/A
24% (*n* = 33)	49% (*n* = 67)	15% (*n* = 21)	8% (*n* = 11)	1% (*n* = 1)	2% (*n* = 4)
In the second month (24th April–22nd May) compared to pre lockdown (before 27th March) has the number of new referrals for secondary mental health services that you provide?					
Significantly increased	Increased	No difference	Decreased	Significantly decreased	N/A
8.% (*n* = 11)	27% (*n* = 37)	37% (*n* = 50)	18% (*n* = 24)	7% (*n* = 10)	3% (*n* = 4)
In the second month (24th April–22nd May) compared to the first month of lockdown (27th March–24th April) has the number of patients attending your secondary mental health service experiencing a relapse of illness?					
Significantly increased	Increased	No difference	Decreased	Significantly decreased	N/A
17% (*n* = 23%)	48% (*n* = 65)	22% (*n* = 30)	4% (*n* = 5)	3% (*n* = 4)	6% (*n* = 8)
In the second month (24th April–22nd May) compared to pre-lockdown (before 27th March) has the number of patients attending your secondary mental health service experiencing a relapse of illness?					
Significantly increased	Increased	No difference	Decreased	Significantly decreased	N/A
13% (*n* = 17)	36% (*n* = 49)	32% (*n* = 43)	8% (*n* = 11)	2% (*n* = 3)	7% (*n* = 10)

The majority of consultants identified that the number of patients already attending services experiencing a relapse of mental illness had also increased/significantly increased during the second month of the lockdown compared to the first (65%, *n* = 88/136). Half of respondents (50%, *n* = 56/133) reported the number of individuals experiencing a relapse was increased compared to before the lockdown. Consultants reported that demand for inpatient beds had increased in the second month compared to the first month (72%, *n* = 78/108). Many services had created alternate pathways for assessments away from the emergency department (ED) (60%, *n* = 81/107). A proportion of consultants (23%, *n* = 21/91) had seen an increase in the number of healthcare worker (HCW) referrals to their service.

Consultants reported an increase/significant increase in the workload for emergency interventions (those that needed to be actioned immediately/within hours) (64%, *n* = 88/137), urgent interventions (those that need to be actioned within 72 hours) (62%, *n* = 83/134) and interventions requiring a response within a month (48%, *n* = 65/135) in the second month of the lockdown compared to the first. Increases in interventions requiring action within 3 months (31%, *n* = 40/135) or after 3 months (21%, *n* = 28/135) were less. See Table 3. Compared to the first month of the lockdown, consultants identified an increase/significant increase in referrals or relapses in self-harm/suicidal ideation (65%, *n* = 85/131), health anxiety (71%, *n* = 91/127), panic attacks/panic disorder (54%, *n* = 69/128), depressive illnesses of new onset (57%, *n* = 74/129) and relapse of unipolar depression (49%, *n* = 62/127) amongst others (Table 3). They also reported an increase/significant increase in presentations of both new onset of psychotic disorders (29%, *n* = 36/125) and relapse of psychotic illness (40%, *n* = 49/124). Consultants reported increased/significantly increased presentations of patients with alcohol abuse (44%, *n* = 55/125), and substance use disorders (39%, *n* = 48/124) in the second month of lockdown compared to the first month.

**Table 3.** Consultants’ perception of the impact of COVID-19 lockdown on team workload and new onset/relapse referrals during second month of the lockdown compared to the first month

In the second month (24th April–22nd May) compared to the first month of lockdown (27th March–24th April) how has the workload in your team changed for the following?					
Intervention	Significantly increased	Increased	No difference	Decreased	Significantly decreased
Emergency interventions (Immediately/within hours)	27% (*n* = 37)	37% (*n* = 51)	30% (*n* = 41)	5% (*n* = 7)	1% (*n* = 1)
Urgent interventions (within 72 hours)	25% (*n* = 33)	37% (*n* = 50)	30% (*n* = 40)	8% (*n* = 11)	0% (*n* = 0)
Interventions usually within 4 weeks	15% (*n* = 20)	33% (*n* = 45)	35% (*n* = 47)	16% (*n* = 21)	1% (*n* = 2)
Interventions usually within 3 months	8% (*n* = 10)	23% (*n* = 30)	50% (n = 65)	16% (*n* = 21)	4% (*n* = 5)
Interventions usually after 3 months	4% (*n* = 5)	17% (*n* = 23)	56% (*n* = 75)	16% (*n* = 21)	7% (*n* = 9)
In your experience, in the second month, compared to the first month of lockdown (27th March–24th April) have you seen any difference in the rate of new referrals or relapses of the following?					
	Significantly increased	Increased	No difference	Decreased	Significantly decreased
Generalised anxiety	26% (*n* = 33)	53% (*n* = 69)	19% (*n* = 24)	1% (*n* = 1)	2% (*n* = 2)
Self harm/ suicidal ideation	13% (*n* = 17)	52% (*n* = 68)	30% (*n* = 39)	5% (*n* = 6)	<1% (*n* = 1)
Depression (new onset)	12% (*n* = 16)	45% (*n* = 58)	40% (*n* = 51)	3% (*n* = 4)	0% (*n* = 0)
Health anxiety	28% (*n* = 36)	43% (*n* = 55)	27% (*n* = 34)	2% (*n* = 2)	0% (*n* = 0)
Panic attacks/panic disorder	12% (*n* = 15)	42% (*n* = 54)	44% (*n* = 56)	2% (*n* = 2)	1% (*n* = 1)
Depression (relapse major depressive disorder)	10% (*n* = 13)	39% (*n* = 49)	48% (*n* = 61)	3% (*n* = 4)	0% (*n* = 0)
Psychotic disorders (relapse)	5% (*n* = 6)	35% (*n* = 43)	59% (*n* = 73)	2% (*n* = 2)	0% (*n* = 0)
Alcohol abuse disorders	10% (*n* = 12)	34% (*n* = 43)	51% (*n* = 64)	5% (*n* = 6)	0% (*n* = 0)
Substance abuse disorders	10% (*n* = 12)	29% (*n* = 36)	58% (*n* = 72)	3% (*n* = 4)	0% (*n* = 0)
Psychotic depression (new onset or relapse)	9% (*n* = 11)	25% (*n* = 32)	65% (*n* = 82)	1% (*n* = 1)	0% (*n* = 0)
Psychotic disorders (new onset)	6% (*n* = 7)	23% (*n* = 29)	70% (*n* = 87)	2% (*n* = 2)	0% (*n* = 0)
Depression (relapse BPAD)	3% (*n* = 4)	22% (*n* = 28)	72% (*n* = 91)	2% (*n* = 3)	0% (*n* = 0)
Mania (relapse BPAD)	5% (*n* = 6)	21% (*n* = 27)	71% (*n* = 89)	2% (*n* = 3)	0% (*n* = 0)
Mania (new onset)	2% (*n* = 3)	17% (*n* = 22)	77% (*n* = 97)	3% (*n* = 4)	0% (*n* = 0)
Eating disorders	5% (*n* = 6)	17% (*n* = 22)	76% (*n* = 97)	2% (*n* = 3)	0% (*n* = 0)
Intellectual disability and autism	12% (*n* = 15)	16% (*n* = 20)	68% (*n* = 86)	3% (*n* = 4)	1% (*n* = 1)

A proportion of consultants (19%, *n* = 25/132) had treated at least one patient with a COVID-19 related neuropsychiatric presentation (delirium/encephalopathy). The majority of consultants had cared for a patient who incorporated COVID-19 into health anxiety (81%, *n* = 110/136), generalised anxiety disorder (72%, *n* = 98/136), but less so for panic disorder (44%, *n* = 60/135). Most consultants had treated at least one patient for whom the consultant felt social isolation was contributing to relapsing or new-onset depressive episodes (81%, *n* = 110/136). See Supplementary Table 1.

### Impact of lockdown on presentations with self-harm/suicidal ideation and new-onset or relapse of psychosis

Less than half of consultants reported that the number of cases with suicidal ideation/self-harm had increased/significantly increased (46%, *n* = 62/136) during the lockdown compared to before the lockdown. See Table 4. Remaining participants predominantly noted no difference in rates (32%, *n* = 44/136). The majority of respondents felt that there was no difference in the lethality of methods used (71%, *n* = 97/137). The majority of consultants had treated at least one patient during the lockdown for whom the consultant felt that social isolation contributed to that person experiencing thoughts of self-harm or suicidal ideation (78%, *n* = 106/136) or contributed to an act of self-harm (64%, *n* = 87/136). Many consultants commented on the importance of monitoring these rates over time.

**Table 4.** Psychiatrists’ experience of effect of lockdown/COVID-19 on self-harm/suicidal ideation factors and psychosis (new onset/relapse)

In your experience, since the lockdown began (27th March–22nd May) compared to before the lockdown has the number of presentations with self-harm/suicidal ideation changed?					
Significantly increased	Increased	No difference	Decreased	Significantly decreased	Not Applicable
9% (*n* = 12)	37% (*n* = 50)	32% (*n* = 44)	12% (*n* = 16)	4% (*n* = 6)	6% (*n* = 8)
In your experience, since the lockdown began (27th March–22nd May) compared to before the lockdown, has the lethality of self-harm methods used by patients changed?					
More lethal methods used		No difference	Less lethal methods used		Not applicable
14% (*n* = 19)		71% (*n* = 97)	3% (*n* = 4)		12% (*n* = 17)
Since the lockdown came into place, have you had experience of at least one patient developing any of the following. Social Isolation factors contributing to thoughts of self-harm/suicidal ideation?					
Yes			No		
78% (*n* = 106)			22% (*n* = 30)		
Since the lockdown came into place, have you had experience of at least one patient developing any of the following. Social Isolation factors contributing to act of deliberate self-harm?					
64% (*n* = 87)			36% (*n* = 49)		
In your experience, since the lockdown began (27th March–22nd May) compared to before the lockdown, has the number of patients with psychosis (new onset or relapse) changed?					
Significantly increased	Increased	No difference	Decreased	Significantly decreased	Not Applicable
9% (*n* = 12)	25% (*n* = 34)	54% (*n* = 73)	1% (*n* = 2)	1% (*n* = 1)	9% (*n* = 12)
In your experience, since the lockdown began (27th March–22nd May) compared to before the lockdown, has the severity of patients presenting with psychosis (new onset or relapse) changed?					
More severe		No difference	Less severe		Not applicable
21% (*n* = 28)		65% (*n* = 86)	2% (*n* = 2)		13% (*n* = 17)
Since the lockdown came into place, have you had experience of at least one patient developing any of the following. COVID-19 being incorporated into delusional/psychotic beliefs?					
Yes			No		
49% (*n* = 66)			51% (*n* = 70)		

Approximately a third of consultants (35%, *n* = 46/134) identified that the number of referrals for new-onset or relapse of psychosis had increased/significantly increased compared to before the lockdown. Majority of remaining participants felt the number of patients either experienced no change (54%, *n* = 73/134). A proportion of respondents (21%, *n* = 28/133) felt these presentations were more severe in nature compared to before the lockdown. Just under half of consultants (49%, *n* = 66/136) had at least one patient incorporate COVID-19 into a delusional belief system.

Opinions as to what factors influenced presentations/relapses for crisis/emergency/urgent presentations, are shown in Table 5. Factors identified included increased isolation (81%, *n* = 109/134), reduced access to usual (face-to-face) secondary mental health supports (79%, *n* = 106/134) and reduced access to community mental health support outside of secondary mental health services (69%, *n* = 92/134).

**Table 5.** COVID-19 associated factors perceived to be influencing presentations

Since the lockdown began (March 27th), in general, how do you feel COVID-19/social distancing may have affected presentations/relapses for crisis/emergency/urgent assessments that you and/or your service provided?	
Answer choice	Applicable (*n* = 134)
Increased isolation	81% (*n* = 109)
Reduced access to usual (face-to-face) secondary mental health supports	79% (*n* = 106)
Reduced access to community mental health support outside of secondary mental health services	69% (*n* = 92)
Reduced access to general practitioner (GP)	57% (*n* = 77)
Increased reliance on drugs and alcohol	46% (*n* = 62)
Violence/ Abuse/ Neglect within home environment	39% (*n* = 52)
Other stressors	34% (*n* = 45)
Social media	24% (*n* = 32)
Reduced access to illicit drugs	20% (*n* = 27)
Made no difference	2% (*n* = 3)

### Impact on mental health act (MHA)

A small proportion [8%, (*n* = 8/102)] of respondents felt that there had been delays in assessment for detention. Reasons cited were gardai being reluctant to become involved as they had no PPE, the team was unable to access a GP and accessing independent consultant opinion. 13% of respondents (*n* = 11/94) felt there were delays in recommendations for detention during the lockdown. Reasons cited were lack of availability of their own GP (57%, *n* = 8/14), availability of GP on call (43%, *n* = 6/14), availability of garda GP (14%, *n* = 2/14), availability of allied admissions (29%, *n* = 4/14), other staff availability (7%, *n* = 1/14) and securing appropriate beds (21%, *n* = 3/14).

### Impact on working day

The majority of consultants (76%, *n* = 98/129) felt that their working day had been affected. Primarily this was due to conducting meetings with telephone/televisual means (81%, *n* = 79/98), providing a mix of telepsychiatry and face-to-face assessments (77%, *n* = 76/98) as outlined in Table 6. No consultants had availed of study leave since the pandemic began. 4% (*n* = 4/98) had been ill with suspected symptoms. 3% (*n* = 3/98) indicated that they had had confirmed COVID-19 infection.

**Table 6.** Alteration in consultant’s working day since onset of pandemic

	Responses	
	%	*n* = 98
Conducting meetings (MDT, management, family) via telephone/videocall	81%	79
Providing a mix of telepsychiatry and face-to-face assessments	78%	76
Conducting supervision of trainees/NCHDs/staff members via telephone/videocall	46%	45
Working remotely	46%	45
Altered timetable due to reconfiguration of services	27%	26
Providing telepsychiatry assessments only	14%	14
Self-isolating – high risk, working remotely	8%	8
Transferred to another setting (please specify)	5%	5
Having to care for someone – not COVID-related	5%	5
Ill with COVID-19 (suspected)	4%	4
Illness (not COVID)	3%	3
Ill with COVID-19 (confirmed)	3%	3
Self-isolating – household with symptoms	2%	2
Having to care for someone – COVID-related	2%	2
Self-isolating – high risk, unable to work	1%	1
Has your service developed a pathway to divert acute presentations from acute hospitals/emergency departments to reduce footfall/infection control in the acute general hospital?			
Yes	No	Not applicable
60% (*n* = 81)	19% (*n* = 26)	21% (*n* = 27)

### Availability of information technology (IT) equipment to conduct duties

The majority of respondents (67%, *n* = 88/132) felt the IT equipment available to them to conduct their duties remotely left them unequipped to conduct some or most/all of these duties. 31% (*n* = 40/132) felt they were fully or well equipped to do most tasks via IT. Respondents felt that patients had variable ability to engage in televisual assessments as opposed to telephone call assessments only [successful/very successful (35%, *n* = 36/103); neither successful or unsuccessful (31%, *n* = 32/103); unsuccessful/very successful (33%, *n* = 34/103)].

### Well-being of psychiatrists

Just over half of consultants (51%, *n* = 61/119) felt that their well-being had decreased/significantly decreased during the pandemic. The majority of remaining participants felt they had noted no noticeable change (49%, *n* = 58/119). The majority of consultants identified their ability to avail of annual leave was decreased/significantly decreased since the onset of the lockdown (54%, *n* = 67/123), the remainder noted no noticeable change (45%, *n* = 55/123). One person noted an increase in availability. The majority of consultants reported that their workloads had increased since the start of the lockdown (62%, *n* = 79/128) or stayed the same (23%, *n* = 30/128). 15% (*n* = 19/128) identified their workloads had decreased. 84% of consultants (*n* = 95/113) anticipated their workloads would increase in coming months. 14% (*n* = 16/113) felt it would stay the same. The remainder felt it would decrease.

### Availability of personal protective equipment (PPE)

The majority of consultants (80%, *n* = 102/128) felt they could access PPE adequately but 13% did not (*n* = 16/128). 7% (*n* = 10/128) did not know. 91% (*n* = 115/126) knew who to raise concerns with about PPE availability in their organisation.

### Thematic analysis

Up to 50 consultants participated in the free-text section of each of the six areas of the questionnaire. Three main themes emerged namely service management, the relationship between patients and healthcare service and the impact of lockdown on consultant’s personal and professional life. This expanded into 6 sub-themes.

### Theme 1: service management

#### Referral process

Consultants described contrasting patterns of GP referrals. Typical examples include GPs referring individuals deemed more suitable for secondary care contrasting with GPs not providing any initial assessment and referring individuals with psychosocial stressors only as no other service available. Illustrative quotes are:

‘A significant issue is that individuals have not been seen by their GP…It would appear that the threshold for onward referral has decreased.’

‘The referrals were more appropriate…less referrals from ED.’

Internal referrals within teams were also affected. Waiting lists for allied health professionals such as occupational therapy and social work all increased. Some patients opted out of receiving telepsychiatry/virtual assessment preferring to be seen face to face once restrictions eased, meaning waiting lists were accumulating. Other patients despite receiving a telepsychiatry assessment still presented to the general hospital, emergency department as they were unhappy with the nature of telepsychiatry. Respite admissions could not be easily arranged, due to infection fears, leading to increased pressure on patients and families in the community. The impression is that some patients were later admitted as inpatients having experienced a more significant relapse. Consultants were reluctant to discharge other patients into the community because of a reduction in community supports/services. Illustrative quotes are:

‘A considerable amount of patients who had been “examined” using a telephone by outpatient CAMHS (Child and Adolescent mental health services) presented to a general paediatric hospital because they were unhappy [with telepsychiatry assessment]…couldn’t talk properly and wanted to be properly examined by a doctor.’

#### Referral pathways

Several consultants described constructive developments whereby patients were able to be seen acutely away for acute hospital settings/emergency departments. Others felt unsupported in developing such pathways. Team under-staffing prior to the lockdown meant any staff leave and team redeployment during the lockdown further impacted on service provision and work-loads of other team members. Staffing deficits were exposed as referral numbers increased. Illustrative quotes include:

‘We tried very hard to get … a pathway to divert acute presentations from acute paediatric hospitals. Unfortunately [management] refused to develop any such pathway, instead sending paediatric patients into a …general paediatric hospital, including accident and emergency.’

‘Lack of alternatives like intensive day hospital services lead to admission. Continuing care unit closed to be converted to Covid ward which had direct impact on inpatient numbers.’

‘Our liaison service is now operating over several pathways - Covid/ non Covid/ ED diversion, and as a team staffed at 30% AVFC (A Vision For Change) we are under severe pressure now that demand is rising.’

### Theme 2: relationship between patients and healthcare service

#### Rapid reduction in availability of community support affected mental health of vulnerable groups

Consultants expressed concerns about the rapid reduction in social structures and supports for patients attending mental health services across the lifespan. Numerous examples were cited including employment, job/financial security and recreational activities. Lack of childcare and primary school, which were identified as protective factors for many children. Lack of access to hobbies/sport, uncertainty around state exams, secondary school and 3rd closure and the switch to online learning was noted to affect older children, teenagers and young adults. Consultants highlighted how difficult the lockdown had been for vulnerable groups of children particularly those who were underprivileged and/or in state care were emphasised and/or with Autistic Spectrum Disorders (ASD), Attention Deficit Hyperactivity Disorders (ADHD), eating disorders and Intellectual Disability. ‘Cocooning’, lack of physical contact with relatives/grandchildren, lack of community nurses calling, carer burden, daycentre closures were frequently identified as factors for older adults.

‘Young people with medical or MH [mental health] vulnerability and young people with social vulnerabilities e.g. in care …and long standing adversity issues have been disproportionately impacted. Community supports routinely used by young people not available. School closures removed a place of safety for many.’

‘BPSD (behavioural and Psychological symptoms of dementia) referral increase may be due to increased family/carer burden during lockdown.’

‘No access to respite, no community support to help care for those with intellectual disabilities.’

#### Challenges in telepsychiatry assessment and provision

Most consultants expressed concerns around the use of telepsychiatry interviews especially for acute assessments. Non-verbal cues could prove difficult to assess and challenges were described in building rapport and performing a complete mental state examination. Although those with chronic medical disorders and adult patients who were stable welcomed the opportunity not to have to attend hospital, many other examples were cited of some patients finding telepsychiatry interviews difficult. These included patients with first episode psychosis reading signals into the video assessment, children with autistic spectrum disorders expressing frustration in interviews and older adults being unable to engage with televisual interviews at all due to dementia or because they were unfamiliar with the medium. Due to the nature of the virtual assessments, the environment where the call was received was also reported as impacting on the assessment. These included the issue of privacy in the home, for example, where the person had not shared their mental health difficulties with family, children being left alone during the interview by parents or having friends in the room. In other cases, ‘cocooning’ meant that family members were prevented from physically helping elderly relatives to assist interviews altogether.

Consultants reported practical issues that affected their ability to provide telepsychiatry interviews – availability of equipment, wi-fi availability/internet connection ability for both consultants and patient and lack of clarity on the safety/availability of suitable forums. Fears were also expressed that telepsychiatry could demedicalise psychiatry and reduce consideration on physical signs/examination in the interview.

‘When someone is acutely unwell it is difficult for them to engage with video/phone assessment.’

‘Many of the patients attending the service have difficulty in engaging [with telepsychiatry]…due to issues with motivation, paranoia, poor digital knowledge.’

### Theme 3: effects on consultant’s personal and professional life

#### Personal life

Consultants described increased stress due to lack of availability of childcare, a blurring of the boundaries between home and working life, with tele-meetings being conducted from home. They also cited concerns of themselves becoming infected and/or infecting vulnerable family members with COVID-19. Lack of face-to-face contact with peers led to feelings of isolation. The risk of burnout amongst consultants was highlighted. Some consultants described increased awareness of their own mental health and the efforts they were taking to address this. A sample of illustrative quotes include:

‘Working from home…long telecons (teleconferences)…to manage the changes in service delivery have caused exhaustion and loss of work life balance.’

‘For consultants with young children at home there has been absolutely no acknowledgment of the additional stress of continuing work/running a service while trying to educate and mind children.’

#### Professional life

Consultants felt an undue amount of care-burden for providing acute services fell to medical/nursing staff compared to colleagues in allied health. Despite fluctuating patient referral numbers, work hours appeared to increase and management responsibilities came to the fore, with focus on rapidly providing new referral pathways, staff education, telepsychiatry provision, adapting premises to remain socially distant, increased administration tasks, for example, trying to organise soft and hardware for remote working for the team. Reduced team time, peer support and reflective practise was highlighted. Concern was expressed these factors would lead to increased burnout in staff. Online peer support/CPD and were cited as supportive measures. A sample of illustrative quotes include:

‘My workload has significantly increased with an expectation that I will be available every weekend 24/7… the stress has been almost intolerable at times.’

‘Increased workload due to providing cover for team members…increased management/service development responsibilities.’

## Discussion

This study describes consultant psychiatrists experience of the impact of the COVID-19 ‘lockdown’ on mental health services over a 2-month period. Consultants reported an initial decrease in presentations in the first month followed by an increase in the second month for both new and pre-attending patients. Respondents also perceived an increase in new and return referrals compared to before the lockdown. The impact of social isolation, reduced access to face-to-face mental health supports and community supports as well as their GP were the main reasons identified. The needs of children and older adults were especially highlighted. Most consultants felt their working day was affected and that their well-being was reduced during the lockdown. The majority knew how to access personal protective equipment (PPE).

Although symptoms such as feeling depressed or anxious may rise during a pandemic in the general population, these experiences can be normal (Qiu *et al.* 2020; Zhang *et al.* 2020). Consultants reported large increases in referrals with mood and anxiety and psychotic disorders, although most diagnostic presentations are reported to have increased during the lockdown. Recent international literature supports this with increased symptoms during the pandemic in individuals with eating disorders (Fernandez-Aranda *et al.* 2020), dementia (Wang *et al*. 2020), ASD (Narzisi *et al.* 2020) and intellectual disability. Those already attending mental health services experiencing a relapse of illness also increased. For example, 40% of consultants reported an increase/significant increase in those with a psychotic disorder. This is especially concerning as we now know that those with a severe mental illness such as those with a schizophrenia spectrum disorder (Nemani *et al.* 2021) are at increased risk of mortality from COVID-19 infection. A relapse of psychosis may affect one’s ability to self-care and follow public health advice.

The impact of physical distancing has meant that face-to-face support services that would normally scaffold individuals in the community were reduced, which may have contributed to why patients appeared to be presenting later when more unwell. Telepsychiatry equipment provision was deemed to be inadequate in the management of emergency/acute presentations as mentioned by several consultants, highlighting the need for an emergency/acute face-to-face response. The need for community based teams to provide emergency based response outside of the emergency department (ED) is long recognised (A Vision for Change, 2006). However only 60% of consultants surveyed stated that alternate pathways to the ED had been created, meaning some patients needing to access emergency mental health care through hospital EDs during pandemic.

Consultants in Ireland perceived an increase in presentations with self-harm and suicidal ideation in the second month of lockdown and to before the lockdown. Consultants perceptions and reports based on data vary. One recent study in Galway reported no increase in the rate of presentations of self-harm/suicidal ideation but noted an increase in the lethality of presentations between 1st March and 31st May 2020 compared to the same time period 2017–2019 (McIntyre *et al.* 2021). There was no evidence of an increase in the actual suicide rate in the Cork area in March–August 2020 (*n* = 15) compared to the same time period in 2019 (*n* = 15), based on real-time surveillance of suicide in Cork (Corcoran 2020). Internationally reports suggest either no rise in suicide rates (Victoria, Australia; England) or a fall (Japan, Norway) in the early months of the pandemic (John *et al.* 2020). During the last economic downturn in Ireland, the suicide rate amongst males rose over 5 years (2008–2012) and was 57% higher than if the pre-recession trend continued. Self-harm rates presenting to hospital were also higher (Corcoran *et al.* 2015). Although a societal ‘pulling together’ phenomenon is described in the early time period following national crisis (Ayers *et al.* 2021), close monitoring will be required to clarify this situation over the coming months and years, especially in the context of rising unemployment levels (McQuinn *et al*. 2020). There is already emerging trends that the rates of domestic violence are increasing (Oireachtas Library & Research Service 2020) and that there are shifts in alcohol consumption patterns to drinking in the home which is especially concerning in households where there are children (O’Dwyer *et al.* 2021). Evidence of increased routine and urgent referrals from September 2020 onwards compared to 2018/2019 in 5 CAMHS services in CHO6 and CHO7 has recently been reported (McNicholas *et al.* 2021). In the longer term, an economic downturn defined by unemployment and financial insecurity may further exacerbate the pressure on mental health services (Roca *et al.* 2020).

Over half of consultants felt their well-being was reduced during the lockdown, putting them at further risk of burnout. A recent systematic review (Howard *et al.* 2019) concluded that psychiatrists, particularly women, suffered from high levels of burnout and psychological distress. It is notable in this study that a proportion of consultants reported an increase in healthcare worker referrals during the lockdown. Individual approaches such as self-care, peer support, Schwartz rounds and Balint groups are helpful at this time, however systematic approaches examining staffing provision are also needed. Over four out of five consultants had access to the correct PPE which was greater than the UK survey in which 60% of all respondents had access. However services should aim for 100% access to PPE given that this is a modifiable factor.

Mental health services in Ireland have experienced decades of under-investment. The proportion of the Irish health budget devoted to mental health has decreased since 2008 and currently stands at 6%, lower than other countries with better developed and better performing mental healthcare systems such as the United Kingdom with budget allocation of 10–13% (Economist Intelligence Unit 2014; Caldas Almeida *et al.* 2015; Department of Health 2017). In the OECD report from 2009, the United Kingdom has double the number of consultant psychiatrists (18/100 000) compared to Ireland at 9/100 000 (OECD, 2009). Clinical staffing levels in Irish MHS were well below levels recommended in A Vision for Change (2006) across the lifespan, for example, in CAMHS services (58.1% of clinical staffing levels), General Adult Community MHS (74.8%) and psychiatry of later life services (60%). These services were already under pressure, experiencing a high level of referrals prior to the pandemic (HSE, 2018), and are vulnerable to rapidly becoming overwhelmed

It is therefore notable that reported referral rates in Ireland were higher when compared to the United Kingdom. This included for referrals deemed urgent/emergency [63% (Ireland) *v.* 43% (U.K.)], referrals needing to be seen within a month (48% *v.* 23%) and for referrals needing to be seen within 3 months (31% *v.* 13%) (Royal College of Psychiatrists, 2021). Although the time-frames are different (our study examined perceptions in the second month of lockdown compared to the first, the UK study examined experience in the 2 weeks prior to the date of study circulation, for example, 17th April to 1st of May), the impression is that Irish mental healthcare services may be seeing a larger increase in referrals compared with the United Kingdom.

Two years following the publication of A Vision For Change (2006), an economic analysis (O’Shea & Kennelly 2008) reported ‘[the government] should set a target of 10 per cent for mental health care expenditure as a proportion of overall health expenditure, to be realised over a 5 year period.’ However, this ambition was not acted on. The recent update to A Vision For Change, Department of Health (2020) highlights the importance of investment (although unspecified) into primary care and mental health. However resourcing community/voluntary services, without resourcing specialist services will result in even greater referrals to secondary care and a lack of capacity within those services to manage these referrals (College of Psychiatrists 2020).

The United Nations has already called for greater investment in MHS to meet the rising need (United Nations 2020; Adhanom 2020) and increased public spending on mental health care leads to individual and societal gains (O’Shea & Kennelly 2008). Therefore the importance of staffing and resourcing our mental health service with increased ring-fenced funding in line with other better performing mental health services internationally to support individuals and their families is imperative, as the pandemic continues. The importance of services reporting on referral data/service needs will also help quantify emerging trends.

This study has a number of strengths and limitations worth considering. The survey had a relatively high participation rate of 32% (compared with the UK (11%) (*n* = 1369/12900). Several free-text sections in the survey offered the opportunity to respondents to provide additional insights beyond the scope of questions asked. Study limitations include that the survey reports subjective perceptions and lacks actual data to investigate referral rates. Those who responded to the survey were self-selecting and had access to the internet which may have introduced selection bias into the results (Bethlehem 2010). Our survey was conducted after the publication of the results of the RCPsych study in the media (Roxby, 2020) which may have biased respondents to this study. Furthermore the bulk of respondents were general adult psychiatrists from urbanised areas of the country which may also have introduced bias in terms of referral rates of different presentations. Consultants from other specialties who did not complete the study may have resulted in their needs not being identified.

## Conclusion

There is now clear evidence that COVID-19 infection leads to psychological sequelae and that existing severe mental illness can lead to increased mortality from COVID-19 (Taquet *et al.* 2021; Nemani *et al.* 2021). Even before the pandemic, we know that those with severe mental illness die at least 15 years younger than the general population (Hjorthøj *et al.* 2017) and that the rate of suicide in Ireland remains a national concern. COVID-19 infection, social isolation, uncertainly surrounding the duration of the pandemic, the fluctuating level of restrictions, the severity of the economic hardship at present and in the future will all impact on the most vulnerable in our society. This and the real risk of health care worker burnout emphasises the critical need for parity of esteem for mental health services with increased and dedicated funding. This is essential if mental health services are to sustainably and effectively respond to the ongoing mental health need.

## Supplementary material

For supplementary material accompanying this paper visit http://dx.doi.org/10.1017/ipm.2021.41. click here to view supplementary material
