# Supplementary material for: Consultant psychiatrists’ experience of the impact of the COVID-19 pandemic on mental health services
Source: Ir J Psychol Med. 2021 Apr 29:1–13. doi: 10.1017/ipm.2021.41 (PMC8503055; doi:10.1017/ipm.2021.41)
Supplement: Supplementary file 1 [file S0790966721000410sup001.docx]

**Supplementary Table 1. Consultant experience of effect of COVID19 infection or beliefs of infection impacting on mental health presentations**

| **Since the lockdown came into place, have you had experience of at least one patient developing any of the following.** | | |
| --- | --- | --- |
|  | Yes | No |
| COVID19 related neuropsychiatry presentation (e.g. delirium/ encephalopathy) | 19%  (N=25) | 81%  (N=107) |
| COVID19 being incorporated into Health Anxiety | 81%  (N=110) | 19%  (N=26) |
| COVID19 being incorporated into Panic Disorder | 44%  (N=60) | 56%  (N=75) |
| COVID19 being incorporated into Generalised Anxiety Disorder | 72%  (N=98) | 28%  (N=38) |
| Social Isolation factors contributing to depressive episode (relapse or new onset) | 81%  (N=110) | 19.12%  (N=26) |
| Anxiety/depression linked with self having/dying from COVID | 48%  (N=64) | 52%  (N=68) |
| Anxiety/ depression linked with family member/ loved one having/dying from COVID | 55%  (N=72) | 45%  (N=59) |
